# Hotspots of Somatic Genetic Variation in Pituitary Neuroendocrine Tumors

**DOI:** 10.3390/cancers15235685

**Published:** 2023-12-01

**Authors:** Mariana Torres-Morán, Alexa L. Franco-Álvarez, Rosa G. Rebollar-Vega, Laura C. Hernández-Ramírez

**Affiliations:** Red de Apoyo a la Investigación, Coordinación de la Investigación Científica, Universidad Nacional Autónoma de México e Instituto Nacional de Ciencias Médicas y Nutrición Salvador Zubirán, Mexico City 14080, Mexico

**Keywords:** genetic driver, mutational hotspot, pituitary neuroendocrine tumor, somatic variant, druggable target

## Abstract

**Simple Summary:**

Mutational hotspots have gained importance as oncological biomarkers in recent years because of their potential as predictors of clinical outcomes and/or therapeutic targets. In addition, they are easily detectable in clinical samples via Sanger or next-generation sequencing (NGS). The role of these genetic defects is less clear in pituitary neuroendocrine tumors (PitNETs), even though the most common genetic drivers of these neoplasms are located within mutational hotspots. Indeed, hotspots in six different genes are of particular importance in this context. Two of them, *USP48* and *SF3B1*, represent very recent and infrequent genetic associations; thus, their clinical relevance remains unclear. For two other genes, *GNAS* and *USP8*, discrepancies exist among studies regarding their associated phenotypes. Finally, the phenotypes associated with *BRAF* and *DICER1* are well defined in other settings, but not yet in sporadic PitNETs. Additional studies are required to assess the potential of these molecular alterations as druggable targets in PitNETs.

**Abstract:**

The most common genetic drivers of pituitary neuroendocrine tumors (PitNETs) lie within mutational hotspots, which are genomic regions where variants tend to cluster. Some of these hotspot defects are unique to PitNETs, while others are associated with additional neoplasms. Hotspot variants in *GNAS* and *USP8* are the most common genetic causes of acromegaly and Cushing’s disease, respectively. Although it has been proposed that these genetic defects could define specific clinical phenotypes, results are highly variable among studies. In contrast, *DICER1* hotspot variants are associated with a familial syndrome of cancer predisposition, and only exceptionally occur as somatic changes. A small number of non-*USP8*-driven corticotropinomas are due to somatic hotspot variants in *USP48* or *BRAF*; the latter is a well-known mutational hotspot in cancer. Finally, somatic variants affecting a hotspot in *SF3B1* have been associated with multiple cancers and, more recently, with prolactinomas. Since the associations of *BRAF*, *USP48*, and *SF3B1* hotspot variants with PitNETs are very recent, their effects on clinical phenotypes are still unknown. Further research is required to fully define the role of these genetic defects as disease biomarkers and therapeutic targets.

## 1. Introduction

Hotspots are genomic regions where variation occurs with a higher frequency than what would be expected by chance [1]. Specific DNA sequences and structures are particularly prone to variation, and cellular processes such as DNA repair and meiosis favor mutational events. For instance, cytosines of CpG or CpNpG sites are preferential targets for methylation, and methylated cytosines are more prone to spontaneous deamination to thymine [2]. Another important mechanism is the GC-biased gene conversion, by which meiotic recombination favors GC-rich over AT-rich alleles [3]. Microsatellites are prone to variation through polymerase slippage and double-stranded DNA breaks (DSBs), which might introduce indels. Other repetitive sequences such as centromeric and subtelomeric regions are at risk for copy number variation and rearrangements [4]. Palindromic AT-rich sequences might also lead to recurrent translocations [5]. Following multiple co-occurring DSBs, chromoplexy and chromothripsis lead to chromosomal rearrangements and the gain or loss of chromosomal regions, respectively. Finally, a process of hypermutation of specific regions known as kataegis results in clusters of single-nucleotide variants (SNVs) biased toward one DNA strand [6].

Genetic changes accumulate in the genome throughout life. The majority of them represent *passenger* variants and only a small fraction are *drivers* for neoplasms [7]. Cancer driver variants, particularly those affecting oncogenes and tumor suppressors, undergo positive clonal selection because they confer advantageous properties to cells, and are thus observed as recurrent genetic defects [1,6]. In cancer genomes, protein-coding regions are enriched in hotspot SNVs and indels [1].

Pituitary neuroendocrine tumors (PitNETs) are usually benign lesions with indolent behavior that display a lower-middle tumor mutation burden, and only occasionally develop features of aggressiveness [8,9]. Multiple hotspots of sequence variation, most of them somatic, have been identified in these tumors. Some of these hotspots are associated exclusively with PitNETs, while others are tumor drivers common to various human neoplasms. Indeed, somatic variants in *GNAS* and *USP8*, which are the most common genetic defects leading to PitNETs, are located in hotspots [10,11,12]. PitNET-associated hotspot variants have attracted interest in recent years as biomarkers because they might determine specific clinical phenotypes. In addition, some of them are known therapeutic targets in other neoplasms, while others represent potentially druggable molecules. We review the most recent information on the association of hotspot variants affecting six different loci with the occurrence of PitNETs, their implications on disease phenotypes, and their potential use as biomarkers and therapeutic targets (Table 1). Genes for which somatic PitNET-associated variants do not cluster in hotspots (such as *MEN1* and *TP53*) are not included in this review. 

## 2. *BRAF*

**Figure 1:**
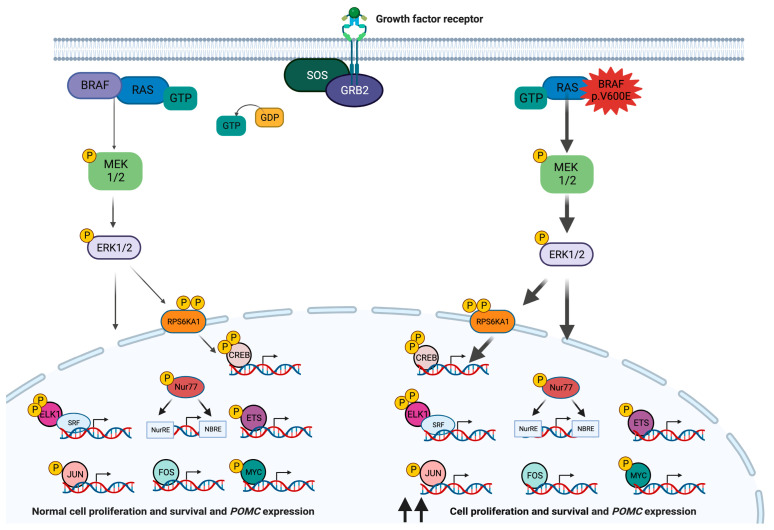
The RAS-RAF-MEK-ERK signaling pathway in corticotroph cells. Under physiological conditions, this pathway is activated in response to the interaction of extracellular ligands such as growth factors, hormones, or cytokines with a tyrosine kinase receptor. The receptor-like growth factor receptor-binding protein 2 (GRB2) binds to the activated receptor and interacts with the proline-rich sequence at the C-terminus of the son of sevenless (SOS) protein to form the receptor-GRB2-SOS complex, which in turn promotes the GTP-mediated activation of RAS. Activated RAS protein binds to and recruits BRAF to the inner side of the cell membrane, where it is phosphorylated by tyrosine kinases. The C-terminal catalytic domain of BRAF interacts with and phosphorylates MEK1 and 2 into their catalytic VIII subregion. In turn, MEK1 and 2 phosphorylate and thus activate ERK1 and 2 (also known as mitogen-activated protein kinases (MAPK) 3 and 1). In addition to phosphorylating cytoplasmic targets, active ERK1 and 2 enter the nucleus and phosphorylate multiple transcription factors, such as ELK1, ETS, FOS, JUN, and MYC, thereby inducing the expression of their target genes. Via the phosphorylation of RPS6KA1, ERK1 and 2 also activate the transcription factor cAMP response element-binding protein (CREB). The activation of this pathway leads to tissue-specific molecular consequences, although in the pituitary gland and in many other tissues it results in increased cell proliferation and survival [18,23,58,59]. In corticotroph cells, this pathway also activates *POMC* transcription, although the membrane receptor triggering this response in physiological conditions and in corticotropinomas remains unclear [40]. The *BRAF* p.V600E variant leads to the overactivation of this signaling pathway.

*Protein kinase* is the most frequently shared domain among cancer-associated proteins, therefore representing a particularly attractive therapeutic target [13]. The isoforms A, B, and C of the highly conserved serine/threonine protein kinase rapidly accelerated fibrosarcoma (RAF) proteins, encoded in humans by three different genes, are among such proteins. C-RAF (also known as RAF-1) was first described in 1985, while A-RAF was discovered in 1986, and B-RAF in 1988 [14,15,16]. The latter is encoded by *BRAF* (7q34, RefSeq NM_001354609.2), a proto-oncogene with preferential expression in neural tissues, and is the most potent activator of the RAS-GTPase (RAS)-RAF-MAPK and ERK kinase (MEK)-extracellular signal-regulated kinase (ERK) signaling pathway (RAS-RAF-MEK-ERK pathway) [17,18,19] (Figure 1). This phosphorylation cascade is involved in the physiological regulation of cellular processes such as proliferation, survival, differentiation, apoptosis, and motility [18].

Germline activating variants affecting either *BRAF* or other members of the RAS-RAF-MEK-ERK pathway are associated with a group of developmental syndromes collectively known as RASopathies [20]. In contrast, the upregulation of this pathway via various mechanisms contributes to tumorigenesis in one-third of human cancers [21]. Specifically, somatic missense activating variants in the glycine-rich loop or the activation segment of the BRAF catalytic domain occur in about 7% of all cancers. At least 90% of such cases, however, are explained by a single defect: c.1799T>A, p.V600E [22,23]. This variant is found in two-thirds of malignant melanomas and papillary thyroid carcinomas (PTCs) and less frequently in colorectal, ovarian, and other types of cancer [22,23,24]. 

The phosphorylation of residues T599 and S602 (UniProt P15056), which flank the variant, is required for BRAF to be recruited to the cell membrane and folded into its active conformation. The p.V600E change destabilizes the inactive conformation of BRAF and promotes its active state, thereby acting as a *phosphomimetic* [25]. This way, *BRAF* p.V600E results in an abnormally active RAS-independent kinase that induces cell proliferation and transformation in vitro and in vivo [22,26,27]. Indeed, *BRAF* variants seem to be mutually exclusive with oncogenic *RAS* defects [22]. In addition to the phosphorylation of the well-known downstream effectors MEK1/2, *BRAF* p.V600E activates NFKB and prevents apoptosis [26,28]. In colorectal cancer, *BRAF* p.V600E has been associated with poor clinical prognosis and chemoresistance, increased microsatellite instability, and a higher mutational load [29]. In addition, quantitation of *BRAF* p.V600E by droplet digital polymerase chain reaction (ddPCR) has been used as a marker for measurable residual disease in hairy cell leukemia [30].

Recent research has demonstrated that BRAF has an important role in the development and terminal differentiation of the anterior pituitary [19,31]. Indeed, patients with cardiofaciocutaneous syndrome (an infrequent RASopathy) caused by activating germline *BRAF* variants may also develop pituitary hormone deficiencies. Although *BRAF* p.V600E has not been clinically identified in this context, its expression in the embryonic anterior pituitary leads to severe hypoplasia in vivo, due to initially accelerated cell proliferation, followed by cell cycle arrest and apoptosis of progenitor cells at later stages [19]. In contrast with this developmental role, somatic *BRAF* p.V600E is associated with the development of papillary craniopharyngiomas (PCPs), which are benign tumors most likely derived from pituitary precursors [32]. Although this hotspot variant is present in 94% of PCPs, it is absent from adamantinomatous craniopharyngiomas, which are usually *CTNNB1*-driven [33,34,35,36,37]. In PCPs, *BRAF* p.V600E is associated with a stable genome and its detection helps to confirm the histopathological diagnosis [34,35,36,37,38]. These tumors usually occur in adults, are not calcified, and have suprasellar location [38,39].

Also recently, somatic *BRAF* p.V600E was identified in 9–10% of cases of non-*USP8*-driven Cushing’s disease (CD) (see Section 6) by two different groups, accounting for a total of 16 cases [40,41]. Subsequent studies failed to identify this defect in other CD cohorts [42,43,44]. The overexpression of *BRAF* p.V600E in mouse corticotropinoma AtT-20 cells led to increased phosphorylation of ERK1/2 and of the transcription factors NUR77, C-JUN, and C-FOS, and consequently, to *Pomc* upregulation. These findings were confirmed on *BRAF* p.V600E-driven corticotropinomas by immunohistochemistry. ACTH secretion was substantially more suppressed by vemurafenib in AtT-20 cells overexpressing *BRAF* p.V600E compared with wild-type *BRAF* [40]. Given the low frequency of this genetic defect, its potential impact on the clinical presentation and response to treatment remains unaddressed. Somatic *BRAF* p.V600E has also been detected in rare cases of posterior pituitary tumors [45]. 

Thanks to the availability of BRAF inhibitors, *BRAF* p.V600E has been exploited as a therapeutic target in many neoplasms. Vemurafenib, dabrafenib, and encorafenib are adenosine triphosphate (ATP)-competitive RAF inhibitors that selectively inhibit BRAF p.V600E, but paradoxically activate RAS-RAF-MEK-ERK signaling in BRAF wild-type tumors, particularly in those with *RAS* activating variants [46,47]. Their many therapeutic applications as single agents or in combination with MEK inhibitors (cobimetinib, binimetinib, and trametinib) and/or other agents including *BRAF* p.V600E-driven colorectal cancer, Erdheim-Chester disease, hematological malignancies, melanoma, non-small-cell lung cancer, and PTC [29,48,49,50,51,52]. 

Individual case reports of PCP treatment with drugs targeting BRAF and/or other RAS-RAF-MEK-ERK components have shown encouraging results [53]. Very recently, a phase 2 clinical trial of combined vemurafenib/cobimetinib treatment in PCP showed a response in 94% of participants, with a median tumor reduction of 91% at 22 months, for progression-free survival of 87 and 58% at 12 and 24 months, respectively [54]. In contrast, BRAF inhibitors have not been evaluated as therapeutic agents for CD in clinical trials. There are, however, three single-case reports of *BRAF* p.V600E positive posterior pituitary tumors (two with confirmed NKX2-1-expression) treated with dabrafenib, either alone [55] or combined with cobimetinib [56] or trametinib [57]. All tumors had recurred after one or more surgeries plus radiotherapy. One patient developed stable disease [57] and two experienced significant tumor regression [55], although the combined therapy resulted in dermatological toxicity.

## 3. *GNAS*

At least ~100 human genes are subjected to genomic imprinting, an epigenetic mechanism that controls gene expression in a parent-of-origin and tissue-specific manner [60]. Using differentially imprinted promoters, one of these genes, *GNAS* (locus of the GNAS complex, 20q13.32), ultimately translates into multiple proteins, namely XLαs, ALEX, NESP55, and Gsα [61,62]. The latter, encoded by a 13-exon reference transcript (NM_000516.7), accounts for the 394-amino-acid α subunit of the heterotrimeric stimulating G protein (P63092-1) [63]. Gsα is translated from the maternal allele in the pituitary, thyroid, and gonads, but depends on biallelic expression in other tissues [64].

**Figure 2:**
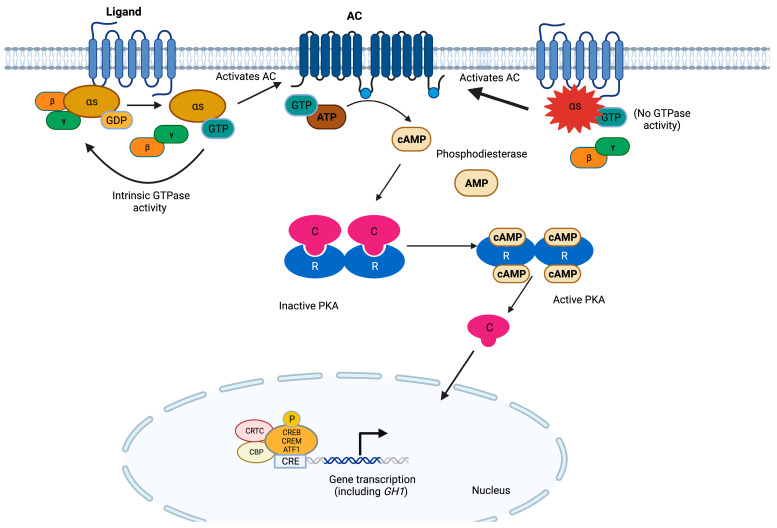
The cAMP pathway in somatotroph cells. G proteins are composed of three subunits, and the α subunit contains high-affinity binding sites for guanine nucleotides. The GDP-bound form binds tightly to βγ and is inactive, whereas the GTP-bound form dissociates from βγ and is the active form. GPCRs cause the activation of G proteins by facilitating the exchange of GTP for GDP on the α subunit, which in turn activates ACs. These enzymes use ATP as a substrate to produce cAMP. The latter binds to the regulatory subunits (R) of PKA, allowing for the release of the catalytic subunits (C). Active PKA catalyzes the serine/threonine phosphorylation of target molecules, including the transcription factors CREB, CRE modulator (CREM), and activating transcription factor 1 (ATF1). In complex with co-activators such as CREB-binding protein (CBP) and members of the cAMP-regulated transcriptional co-activators (CRTC), these transcription factors bind the 8 bp palindromic sequence known as cAMP response element (CRE) in the promoter region of target genes to increase their transcription. In somatotrophs, the GH-releasing hormone receptor (GHRHR) is the main GPCR activating this pathway, promoting both cell proliferation and *GH* transcription [66,67,68,69,121]. *GNAS* hotspot variants result in the constitutive activation of this pathway.

At the molecular level, guanine nucleotide-binding proteins (G proteins) function as information transducers between the cell-membrane-bound G-protein-coupled receptors (GPCRs) and their effectors, thereby regulating the production of second messengers [65]. G proteins are composed of α, β, and γ subunits (encoded by different genes) and form a complex that binds GPCRs [66]. Gsα is made of a C-terminal RAS-like guanosine triphosphatase (GTPase) that also functions as an interaction site for the β and γ subunits and an N-terminal helicoidal domain [67]. A nucleotide binding cleft exists in between those two domains, which binds guanosine diphosphate (GDP) while the GPCR is inactive. Following GPCR activation through ligand binding, Gsα exchanges GDP for guanosine triphosphate (GTP) and dissociates from the βγ dimer and the receptor, thereby allowing for the GNAS-dependent activation of adenylyl cyclases (ACs) [68,69]. ACs in turn catalyze the synthesis of cyclic 3′,5′-adenosine monophosphate (cAMP), which then activates downstream signaling pathways [66]. This activation cycle is negatively regulated by the intrinsic GTPase activity of Gsα, which prevents the continued activation of downstream effectors [68] (Figure 2). The effects of multiple hormones greatly depend on cAMP, and the specificity of the cellular responses elicited by this second messenger is determined in a tissue-specific manner [70].

Missense *GNAS* variants affecting residues R201 (namely p.R201C, p.R201S, and p.R201H), and G227 (p.G227R, p.G227L, and p.G227K) of GNAS have been described in endocrine tumors and other human neoplasms. They have been found as somatic changes in somatotropinomas (4.4–59.5%), non-functioning PitNETs (7–10%), corticotropinomas (6%), autonomous thyroid adenomas (5%) and thyroid cancer (13% of PTC and up to 4% of follicular tumors), and occasionally, in ovarian and testicular Leydig cell tumors, prolactinomas, adrenocortical adenomas, pheochromocytomas, paragangliomas, parathyroid adenomas, and in patients with multiple endocrine tumors [10,71,72,73,74,75,76,77,78,79,80,81,82,83,84,85,86,87,88,89,90,91,92,93,94,95,96,97,98]. These variants have also been found in non-endocrine malignant neoplasms, such as pancreatic, colorectal, and lung adenocarcinomas, as well as in hepatocellular carcinomas [99,100,101,102].

*GNAS* variants also underlie the McCune–Albright syndrome (MAS, MIM #174800), a rare condition with sporadic presentation characterized by genetic mosaicism due to early postzygotic *GNAS* hotspot defects [103,104]. The diagnosis is established in the presence of two or more of the classic MAS features: polyostotic fibrous dysplasia, *café-au-lait* skin spots, and endocrine hyperfunction (gonadotropin-independent precocious puberty, hyperthyroidism, early-onset Cushing’s syndrome, and PitNETs, usually GH or GH and prolactin-secreting, among others) [105]. Ninety-five percent of MAS cases are due to variants in R201, while only 5% are caused by variants in Q227 [106,107,108]. The phenotype is determined by genomic imprinting and the disease severity correlates with the degree of mosaicism, meaning that the clinical presentation depends on the time of appearance of the *GNAS* variant during embryogenesis [109]. 

*GNAS* hotspot variants cause the loss of protein function that results in increased activity of the cAMP signaling pathway, by (1) stabilizing Gsα in its active conformation, thereby mimicking the effect of extracellular growth factors by stimulating ACs, and (2) inhibiting GTPase activity and causing a constitutive activation of ACs [10,110]. For these reasons, these *GNAS* defects are often referred to as activating variants or *gsp* oncogene [10]. Restoring the GTPase activity of *GNAS* is an attractive therapeutic target, although drugs with this specific effect have not been reported yet. In contrast, non-hotspot loss-of-function (LOF) *GNAS* variants cause Albright’s hereditary osteodystrophy [111].

The clinical consequences of *GNAS* variants have been thoroughly studied in somatotropinomas. Some studies have defined a particular *GNAS*-associated phenotype, with patients usually being older and presenting significantly smaller tumors associated with low serum GH or IGF1 levels [71,89,96,97]. Other studies have described *GNAS*-driven tumors as having a slow growth rate and a better response to pharmacological or surgical treatment compared with wild-type tumors [96,98,112]. These tumors are usually of the densely granulated subtype at the histopathological examination [113]. Differences in age, sex, and other clinical characteristics have been suggested by some studies [89,114,115]. At the molecular level, *GNAS* hotspot variants define a distinctive subgroup of somatotropinomas that display hypomethylation, limited chromosomal alterations, and activation of the GPCR pathway, although results vary among studies [98,116,117]. In both sporadic and MAS-related somatotropinomas, *GNAS* variants almost always affect the maternal allele [118]. While wild-type somatotropinomas often display relaxation of the paternal imprinting, this phenomenon is infrequent in tumors carrying *GNAS* variants [97,119,120]. The relaxation of *GNAS* imprinting correlates with lower *GNAS*, *SSTR2*, and *AIP* expression, suggesting a possibly reduced response to somatostatin receptor ligands [97].

## 4. *DICER1*

The DICER1 syndrome (MIM #601200) is an autosomal dominant condition of tumor predisposition that encompasses otherwise infrequent dysembryonic tumors, such as pleuropulmonary blastoma (PPB), cystic nephroma (CN), ovarian sex cord stromal tumor, nasal chondromesenchymal hamartoma, ciliary body and cerebral medulloepitheliomas, anaplastic kidney sarcoma, pineoblastoma, embryonal rhabdomyosarcoma (ERMS), and pituitary blastoma (PitB) [122]. Other associated neoplasms are Wilms tumor (WT), juvenile hamartomatous intestinal polyps, and differentiated thyroid carcinoma, as well as benign lesions such as multinodular goiter and pulmonary cysts.

This syndrome presents usually at an early age and occasionally in young adults and is caused in most cases by germline heterozygous LOF *DICER1* (14q32.13) variants that appear de novo in 10–20% of cases [123,124,125,126]. Ten percent of cases are due to somatic mosaicism for *DICER1* variants, which has been associated with earlier disease onset, more *DICER1*-associated tumors, and a distinctive presentation known as GLOW syndrome (global developmental delay, lung cysts, overgrowth, and Wilms tumor) [125,127,128].

**Figure 3:**
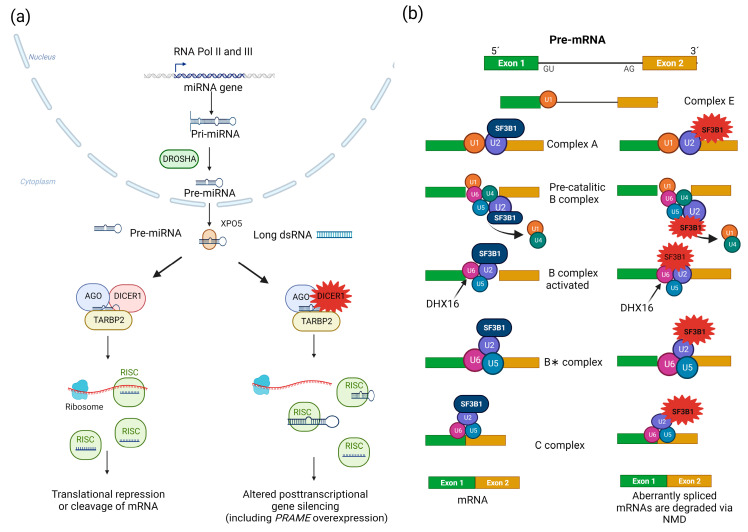
RNA processing pathways involved in PitNETs. (**a**) Biogenesis of small RNAs. In the nucleus, RNA polymerases II and III (RNA Pol II and III) generate primary miRNA transcripts (pri-miRNAs) from miRNA-encoding genes, which are then processed by the microprocessor complex, including the DROSHA RNaseIII. This initial step renders ~60-nucleotide-long hairpin-folded pre-miRNAs, which are in turn exported to the cytoplasm via exportin 5 (XPO5)/Ran-GTP. In the cytoplasm, DICER1 cleaves pre-miRNAs and long dsRNAs into mature miRNAs and siRNAs, respectively, both of which are 20–22 nucleotide-long double-stranded RNAs. The DICER1-dsRNA complex is then bound by a member of the AGO protein family (AGO2 is the best characterized of them) and TARBP2 to form the RISC-loading complex. This complex in turn loads dsRNAs into the RISC, which is required to produce single-stranded small RNAs that serve as a guide to recognize complementary RNA sequences (located in the 3′ untranslated region of mRNAs). The small RNA-loaded RISC can either block translation and promote degradation or directly cleave (via AGO proteins) target mRNAs. Additional roles for DICER1 in the responses to DNA damage (nuclear) and viral infections (cytoplasmic) have recently been described. In PitBs, this abnormal repertoire of small RNAs results in *PRAME* dysregulation, among other transcriptional alterations [131,133,155,156,157]. *DICER1* variants result in abnormal processing of small RNAs, thereby impairing their ability to regulate gene expression. (**b**) Processing of mRNAs by the spliceosome. The spliceosome is a large complex of snRNPs and other proteins that carries out the removal of introns and the ligation of exons from mRNA precursors (pre-mRNAs), rendering mature mRNAs. Two types of spliceosomes, U2-dependent and U12-dependent, are recognized in eukaryotes, the former being the predominant one. The U2-dependent spliceosome is composed of U1, U2, U5, and U4/U6 snRNP, as well as other proteins. This process beings when the U1 snRNP binds to the 5′ SS to form the E complex. Then, the non-ribonucleoprotein complex components SF1, U2AF2, and U2AF1 bind the BS (18–40 nucleotides upstream from the 3′ SS), the polypyrimidine tract (a sequence immediately downstream from the BS), and an AG dinucleotide at the intron-exon junction, respectively. The U2 snRNP in turn replaces SF1, forming the A complex, and the U5, and U4/U6 snRNPs are then recruited to form the precatalytic B complex. Rearrangements in RNA–RNA and RNA–protein interactions ultimately lead to dissociation of the U1 and U4 snRNPs, thereby producing the active B complex. The latter is activated by the pre-mRNA-splicing factor ATP-dependent RNA helicase DHX16, thereby generating the B∗ complex, which catalyzes the first step of splicing. The C complex is then formed, triggering the second step of splicing. Finally, the spliceosome is removed and recycled. *SF3B1* hotspot variants lead to the use of cryptic pre-mRNA 3′ SSs, and aberrantly spliced mRNAs are degraded via NMD [158,159,160,161,162,163,164]. The repertoire of aberrantly spliced mRNAs involved in lactotroph tumorigenesis remains unknown.

The 29-exon *DICER1* canonical transcript (NM_030621.4) encodes a widely expressed 1922 amino acid cytoplasmic enzyme (Q9UPY3-1) composed, from N- to C-terminal, of a helicase domain, a domain of unknown function (DUF283), a platform domain, a P-element-induced whimpy tested (PIWI)-Argonaute (AGO)-Zwille (PAZ) domain, a connector domain, the class 3 ribonuclease (RNase III) a and b domains, and a double-stranded RNA (dsRNA)-binding domain [129]. DICER1 plays a crucial role in the processing of small RNAs, which are the RNA species involved in gene silencing. It first cleaves pre-miRNAs and long dsRNA substrates into mature microRNAs (miRNAs) and small interfering RNAs (siRNAs), respectively [130,131]. Then, DICER1 participates in the loading of siRNAs and miRNAs onto the RNA-induced silencing complex (RISC), composed of DICER1, an AGO protein, and the RISC-loading complex subunit transactivating response RNA-binding protein (TARBP2) [132]. The AGO protein selects a strand of the small RNA as a guide, which in turn directs the small RNA-bound RISC complex toward complementary messenger RNA (mRNA) sequences. The mRNA targets are then either cleaved by AGO (RNA interference) or translationally repressed and directed to degradation (miRNA-mediated gene silencing); the latter mechanism predominates in mammalian cells [133] (Figure 3).

Most individuals carrying germline *DICER1* variants also harbor somatic second hits, which in most cases are missense changes and rarely loss of heterozygosity (LOH) [128]. Moreover, somatic deleterious *DICER1* variants have been reported in the presence or absence of germline defects in patients with PPB, CN, WT, non-epithelial ovarian tumors, cervical ERMS, PitB, prostate carcinoma, pineoblastoma, differentiated thyroid carcinoma, and testicular germ cell tumors [134,135,136,137,138,139,140,141,142,143,144]. Different to germline variants, which are usually truncating and are not clustered in hotspots, most mosaic and somatic variants occurring isolated or as second hits are missense and located within the RNase IIIb domain [128,141,145]. 

Nineteen out of the twenty PitBs genotyped so far were due to LOF *DICER1* variants, although it is not clear if any cases were caused by somatic defects [126,146,147,148]. These tumors usually affect neonates or infants, but one case diagnosed in childhood and one presenting in young adulthood have been reported [146,147,148]. These extremely rare and poorly differentiated anterior pituitary neoplasms with a so-called *oncofetal* molecular signature usually express ACTH and may present clinically silently or as CD [141,149,150]. Nine of these patients died during infancy or childhood due to tumor-related complications [147,148]. Because PitB is considered a pathognomonic lesion of the DICER1 syndrome, its diagnosis should prompt germline *DICER1* screening and genetic counseling [126].

RNAse IIIb variants affect metal ion binding and adjacent amino acids, specifically 1705, 1709, 1809, 1810, or 1813, which are therefore considered missense hotspots [122,131]. Second somatic variants outside the hotspot as well as LOH have also been described in patients with somatic mosaicism for RNAse IIIb variants [125,144]. The abnormal RNase IIIb cleaves 5′-derived miRNAs from the pre-miRNA hairpin loops inefficiently, causing retention of pre-miRNA loop sequences and leading to reduced expression of 5′-derived mature miRNAs and predominance of 3′-derived pre-miRNAs [144]. The oncogenic capacity of the biased pre-miRNA repertoire seems to depend on the cellular and developmental setting [145].

In PitB and other *DICER1*-associated tumors, this abnormal miRNA repertoire leads to the overexpression of the preferentially expressed antigen in the melanoma gene (*PRAME*) [150,151]. PRAME is a member of the retinoic acid receptor (RAR) signaling pathway that may act as an oncogene or as a tumor suppressor depending on the cellular context. This protein is highly expressed in melanoma and other malignancies, but not in most normal tissues, except for testes, and, at lower levels, ovaries, adrenals, and endometrium [152]. Aside from RAR, the WNT, NOTCH, and PI3K signaling pathways are also activated in PitB, although the specific pro-tumorigenic downstream effects of *PRAME* overexpression remain unclear [150,151]. *PRAME* overexpression has recently been explored as a potential therapeutic target for immunotherapy in various neoplasms, although not yet in patients with *DICER1* LOF [152].

Aside from its role as a tumor driver, reduced *DICER1* expression due to haploinsufficiency or other mechanisms correlates with bad outcomes in multiple types of cancer [131]. In these tumors, unprocessed pre-miRNAs are degraded by the endonuclease complex TSN-TSNAX. Pharmacological or shRNA-mediated inhibition of this complex facilitates the restoration of miRNA levels by DICER1 in vitro, making it a potential therapeutic target [153,154]. This strategy, however, has not yet been explored in tumors carrying *DICER1* hotspot variants.

## 5. *SF3B1*

Using genome sequencing in 21 patients and targeted genotyping by ddPCR in the rest, a recurrent missense somatic variant (c.1874G>A, p.R625H) in the splicing factor 3B subunit 1 gene (*SF3B1*, 2q33.1, NM_012433.4) was identified in 20% of prolactinomas of a single cohort of 227 cases [165]. When 154 PitNETs of other types were tested, this variant was only found in 6% of cases, all of them staining positive for prolactin. Individuals carrying *SF3B1* p.R625H displayed significantly higher prolactin levels and a shorter progression-free survival, compared with *SF3B1* wild-type cases. A recent Sanger sequencing-based study identified the same variant and an additional missense variant in the same residue (c.1873C>T, p.R625C) in 7 out of 282 prolactinomas analyzed (2.5%) [166]. Interestingly, 50% of metastatic prolactinomas carried *SF3B1* hotspot defects. In line with the earlier findings, *SF3B1* variants were associated with a larger tumor size and increased mortality, but also with a higher Ki67 index and a need for more therapeutic interventions.

*SF3B1* encodes a component of the U2 small nuclear ribonucleoprotein (snRNP) complex and is therefore a component of the pre-mRNA splicing machinery. SF3B1 is involved in 3′ acceptor splice site (SS) recognition, as well as in recruiting other U2 snRNP subunits to the branch point (BP) of pre-mRNAs via interaction with the BP and U2AF2 [167] (Figure 3). The canonical form of SF3B1 (O75533-1) is a 1304-amino-acid protein containing an unstructured N-terminal region, while the C-terminal two-thirds of the protein constitute a huntingtin, elongation factor 3, regulatory A subunit of protein phosphatase 2A, and TOR1 (HEAT) domain, composed of 20 tandem repeats [158]. 

Recurrent somatic variants in hotspots within the fifth and ninth HEAT repeats have been found in myelodysplastic syndrome, chronic myelomonocytic leukemia, acute myeloid leukemia, myeloproliferative neoplasms, primary myelofibrosis, chronic lymphocytic leukemia, breast cancer, pancreatic ductal adenocarcinoma, uveal, mucosal, and cutaneous melanoma, and prostate cancer [159,168,169,170,171,172,173,174,175]. Aberrant splicing is a well-known tumorigenic mechanism, and indeed, abnormal splicing patterns have been demonstrated in neoplasms carrying *SF3B1* hotspot variants in some [168,169], although not all studies [173]. In prolactinomas, p.R625H (in the fifth HEAT repeat) leads to aberrant splicing of estrogen-related receptor gamma (*ESRRG*) mRNA, resulting in stronger interaction with the pituitary-specific positive transcription factor 1 (POU1F1) and excessive prolactin secretion [165]. This variant also causes aberrant splicing and downregulation of *DLG1* in human prolactinomas and rat somatotropinoma GH3 cells. In the latter, the variant causes an epithelial–mesenchymal transition phenotype [176].

The SF3B1 residues involved in neoplasia are crucial for maintaining the tertiary structure of the protein. Their substitution induces conformational changes in the HEAT domain that hamper the interaction of SF3B1 with the BP and with other U2 snRNP components [158]. This results in SF3B1 recognizing alternative BPs upstream of the canonical ones, leading to the use of cryptic pre-mRNA 3′ SSs that are less dependent on U2AF2 and thus promoting the production of aberrantly spliced mRNAs [159,160,161]. The final consequence is downregulation of the affected transcripts because approximately 50% of the aberrant mRNAs undergo nonsense-mediated decay (NMD) [160]. Interestingly, a previous study showed that multiple components of the splicing machinery were dysregulated in PitNETs, although this finding was not specific for corticotropinomas [177]. 

The significance of *SF3B1* variants for clinical prognosis in neoplasms remains unclear, but they could represent a druggable target [170]. *SF3B1* silencing in breast cancer cell lines inhibited aberrant splicing, reducing cell proliferation, migration, and invasion, suggesting a potential antineoplastic role for SF3B1 inhibitors [178]. Examples of such compounds are the natural products pladienolide B, spliceostatin A, herboxidiene, and sudemycin, not available for clinical use, and the synthetic derivatives of pladienolide B, E7107 and H3B-8800, which have been tested in phase I clinical trials [179]. Intravenous E7107 showed low activity and severe ophthalmologic toxicity when used against solid tumors [180]. Oral H3B-8800, however, has shown moderate efficacy and an acceptable profile of adverse effects in patients with myeloid neoplasms [181]. Interestingly, pladienolide B reduced cell proliferation, viability, and hormone secretion in GH3 and AtT-20 (mouse corticotropinoma-derived) cell lines, as well as in primary PitNET cell cultures [177]. Further studies are required to fully assess the effectiveness of this drug in tumors carrying *SF3B1* hotspot variants.

## 6. *USP8*

Research published over the last eight years has established that somatic defects in codons 718–720 of *USP8* (exon 14 in NM_005154.5) are the most frequent genetic cause of CD, being found in 21–62% of corticotropinomas [11,12,43,182,183,184,185,186,187,188,189,190,191,192,193,194,195]. Seventeen different pathogenic or likely pathogenic variants at the protein level have been reported so far (reviewed in [196]). *USP8* (15q21.2) encodes the 1118-amino-acid ubiquitin carboxyl-terminal hydrolase 8 (P40818-1), which belongs to the family of deubiquitinases (DUBs) [63]. Ubiquitination is one of the most frequent forms of protein posttranslational modifications and plays a major role in the protein quality control system by promoting proteasomal or lysosomal degradation, thereby controlling protein turnover. DUBs remove ubiquitin molecules from other proteins and are involved in the generation and maintenance of free ubiquitin monomers [197].

**Figure 4:**
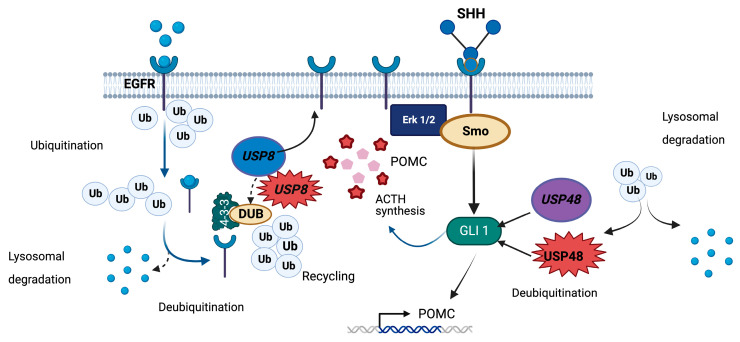
Roles of USP8 and USP48 in deubiquitination in corticotroph cells. DUBs counteract ubiquitination of specific targets, thereby preventing their proteasomal or lysosomal degradation [212]. Upon ligand binding, EGFR is internalized, and then ubiquitinated, and directed for degradation by the lysosomal pathway. USP8 deubiquitinates EGFR both at the cell membrane and in the lysosomes, reducing its degradation and favoring its recycling. In corticotroph cells, EGFR signaling promotes cell proliferation, *POMC* expression, and ACTH secretion. Another important regulator of corticotroph tumorigenesis is the SHH pathway. Its effector, GLI1, is a substrate for USP48 that under physiological conditions leads to increased *POMC* expression. Another USP48 target, NFKB, has the opposite effect [40,41,197,201]. *USP8* and *USP48* hotspot variants associated with corticotropinomas lead to enhanced EGFR and GLI signaling, respectively. *USP48* variants also inhibit the function of NFKB. Additional effects of hotspot variants affecting these DUBs are still incompletely described.

The mutational hotspot implicated in CD lies within the USP8 14-3-3 interacting motif (residues 715–720), which is crucial for maintaining USP8 protein integrity. The CD-associated variants lead to the loss of the 14-3-3 interaction and cleavage of USP8 just upstream of the interacting site, resulting in a C-terminal 40 kDa protein fragment with an enhanced DUB activity [12]. The epidermal growth factor receptor (EGFR) is a particularly affected DUB target in this setting, because USP8 indirectly regulates its deubiquitination both at the cell membrane and at the early endosomes, the latter via interaction with the endosomal sorting complex-III required for transport (ESCRT-III) [197,198,199,200]. Specifically, while EGF signaling promotes the ubiquitination of the ESCRT-III member CHMP1B, which is essential for EGFR degradation, USP8 counteracts this effect [201]. By deubiquitinating CHMP1B, USP8 also indirectly regulates the deubiquitination of EGFR in the early endosomes [201] Indeed, *USP8* activating variants result in increased EGFR recycling, which in turn leads to a rise in *POMC* transcription [11,12,183] (Figure 4).

The role of this tyrosine kinase receptor as a potent inducer of POMC expression and ACTH secretion with apparent autocrine/paracrine regulation in corticotropinomas has been thoroughly characterized [202]. Nevertheless, *USP8* hotspot variants seem to also impact other signaling pathways, including the downregulation of genes involved in protein degradation and cell-to-cell junction [193]. USP8 also deubiquitinates other proteins, such as ERBB2, ERBB3, MET, LRIG1, EPS15, HGS, STAM, STAM2, CHMP proteins, CFLAR, NOTCH1, GJA1, and AKT [198,203].

A recent RNA sequencing study found higher expression of *POMC, CDC25A* and *MAPK4* in tumors carrying *USP8* variants; these findings point toward an enhanced secretory and proliferative potential. In contrast, *USP8* wild-type tumors expressed higher levels of *CCND2*, *CDK6*, and *CDKN1B,* while the expression of *EGFR* and *USP8* did not differ among groups [188]. A different study found reduced immunoreactivity for CDKN1B (in concordance with the transcriptomic study) as well as increased immunoreactivity for HSP90 and pCREB in corticotropinomas with *USP8* variants [190]. The potential clinical significance of these findings requires further exploration.

From the published data, it is clear that *USP8* variants are more frequent in women and in younger adults [11,12,41,43,182,183,184,186,187,188,189,190,191,192,193,194,195]. In pediatric CD, this defect is less common (0–14%) and is found in teenagers more often than in younger children [44,194]. A possible explanation might be that hereditary causes of CD are more common in children than in adults, and patients carrying germline drivers of CD are usually *USP8* wild-type [44].

A favorable phenotype was originally proposed for corticotropinomas carrying *USP8* variants because the first studies reported that they were smaller than their wild-type counterparts and because they almost always present with overt CD and not as silent corticotropinomas [11,12,183]. In addition, two groups reported higher clinical remission rates in cases with *USP8* variants [183,189]. Along these lines, *USP8* variants are less frequently found in Crooke’s cell adenomas than in other corticotropinomas and do not seem to drive Nelson’s syndrome [183,204]. Also, tumors with *USP8* variants display increased immunoreactivity for SST5 and MGMT, which could favor the response to medical treatment [183,191]. Indeed, corticotropinomas carrying the p.P720R displayed an increased response to pasireotide in vitro [205]. USP8 itself might be a druggable target, since USP8 inhibitors reduced cell proliferation and ACTH secretion in mouse corticotropinoma-derived AtT-20 cells [206].

In contrast, other studies found that *USP8* variants were found in larger tumors [189,192,195,204] and were associated with higher recurrence rates [185,186,195] and/or earlier recurrences [11,186]. Other effects on the clinical phenotype, perhaps not related to tumor aggressiveness, have also been explored. Among them, an association was found with higher urinary free cortisol, with or without higher serum cortisol suppression with low-dose dexamethasone [186,204]. This genetic defect has also been associated with enhanced cortisol and ACTH responses with the high-dose dexamethasone suppression test and the desmopressin stimulation test, as well as with lower plasma ACTH [11,42,183,207].

While the effects of *USP8* hotspot variants have been thoroughly explored at the somatic level, their systemic consequences are less well known. Only one case of a germline *USP8* hotspot has been described, in a female individual with a history of recurrent pediatric CD, developmental delay, dysmorphic features, and other medical issues [208]. The variant (p.S719P), had been detected previously as a somatic change in corticotropinomas and was found to cause gain-of-function in vitro [183].

USP8 has been investigated as a pharmacological target, either indirectly through its associated activation of the EGFR pathway, or via direct inhibition. Regarding the former, the EGFR inhibitor gefitinib has shown potential as a treatment for CD in vitro and in vivo [209]. In terms of direct inhibition, compounds specifically targeting USP8 such as DUBs-IN-2 and RA-9 have shown some therapeutic potential in vitro [206,210,211]. These drugs, however, are not yet available for clinical use.

## 7. *USP48*

The ubiquitin-specific protease 48, encoded by the *USP48* gene (1p36.12), is another DUB that has recently been implicated in the pathogenesis of CD. Recurrent somatic missense variants affecting a hotspot in residue 415 (Q86UV5-1, exon 10 in NM_032236.8) of *USP48* (p.M415I or p.M415V) have been identified in 4–23% of corticotropinomas negative for *USP8* variants in the general population and in only 1% of *USP8* wild-type pediatric corticotropinomas [40,41,43,44,195,213]. 

These variants lie within the peptidase domain of the protein, specifically in its catalytic “palm”, but do not affect its expression [40]. The abnormal protein displays increased DUB activity in vitro, thus leading to reduced degradation of USP48 substrates, such as histone H2A and GLI1 [41]. *USP48* variants result in increased POMC transcription by at least two possible mechanisms. The sonic hedgehog (SHH) pathway effector GLI1, which is overexpressed in this setting, potentiates the stimulatory effect of CRH on the *POMC* promoter [41]. Interestingly, SMO, another SHH member, is a substrate of USP8 [214]. In addition, *USP48* variants also lead to the inhibition of NFKB, probably via enhanced stabilization of its RELA subunit, thereby blunting its effect as a *POMC* negative transcriptional regulator [40] (Figure 4).

Corticotropinomas carrying *USP48* hotspot defects were significantly smaller in one study and displayed a higher rate of cavernous sinus invasion in a different cohort, compared with *USP48* wild-type tumors [41,43]. A third study found no significant differences in the clinical phenotype [40]. Compared with the wild type, these tumors express lower levels of *CCND2* at the mRNA level and of *CDKN1B* at the mRNA and protein levels [213]. Unfortunately, the clinical significance of *USP48* hotspot variants has been explored in only a small number of patients, given its rarity, and thus requires further assessment. Just like USP8, USP48 could also be directly targeted by DUB inhibitors (DUBs-IN), although there are no data in the literature on USP48-specific compounds [215].

## 8. Conclusions and Future Directions

Hotspot variants associated with PitNETs have been proposed as potential disease biomarkers by multiple groups. Although *GNAS* and *USP8* might define specific clinical phenotypes, results are highly variable among studies. In contrast, *DICER1* variants are clearly associated with an aggressive phenotype, but they only exceptionally occur as somatic changes. Since *BRAF*, *USP48*, and *SF3B1* hotspot variants have only recently been identified in PitNETs, their associated clinical pictures are still unknown. All these variants are easily detectable by Sanger sequencing or NGS techniques, which might become part of routine diagnostic tumor assessments in the near future. Further preclinical and clinical research protocols are required to test the efficacy and safety of compounds directed toward these molecular alterations in PitNETs. 

## Figures and Tables

**Table 1:** Summary of the molecular and clinical implications of hotspot genetic defects for pituitary tumorigenesis.

Gene	Hotspot Defect	Effect on Protein	PitNET Subtypes Affected	Clinical Phenotype	Pharmacological Agents Targeting the Hotspot
*BRAF*	Somatic p.V600E	Destabilization of the inactive conformation of BRAF, promoting its activation	Corticotropinomas	Unclear (very infrequent genetic defect in PitNETs)	Vemurafenib, dabrafenib, and encorafenib, as monotherapy or combined with MEK inhibitors (in clinical use for other tumor types)
*GNAS*	Somatic missense variants affecting residues 201 or 227	Stabilization of Gsα in its active conformation and inhibition of its GTPase activity	Somatotropinomas. Less frequently: NF-PitNETs and corticotropinomas	Older patients. Controversial: low GH or IGF1, small tumors with slow growth rate, densely granulated somatotropinomas	None reported
*DICER1*	Germline and (or?) * somatic variants affecting residues 1705, 1709, 1809, 1810, or 1813	Loss of RNase IIIb activity	PitBs	CD in infants, neonates, or, less frequently, in childhood and young adulthood. Rarely: silent corticotropinomas. Large, aggressive, and poorly differentiated tumors with an oncofetal signature. Presentation as isolated tumors or as part of DICER1 syndrome	Not reported. Indirect approach: pharmacological inhibition of the endonuclease complex TSN-TSNAX (not available for clinical use)
*SF3B1*	Somatic p.R625H and p.R625C	Impaired interaction with the BP and with other U2 snRNP components	Prolactinomas	High serum prolactin, large tumors with increased Ki67 index, increased progression-free survival and mortality, and requirement for multiple treatments. Occasionally: metastatic PitNETs	H3B-8800 and pladienolide B (not available for clinical use)
*USP8*	Somatic variants affecting residues 718–720	Impaired interaction with 14-3-3, proteolytic cleavage, and enhanced deubiquitinase activity	Corticotropinomas	CD in young adults and teenagers, most frequently women, increased SST5 and MGMT, rarely Crooke’s cell adenomas, not associated with Nelson’s syndrome. Controversial: effects on tumor size, hormone secretion, response to dynamic tests, and remission/recurrence rates	DUBs-IN-2 and RA-9 (not available for clinical use). Indirect: gefitinib (in clinical use for other tumor types, under research for corticotropinomas)
*USP48*	Somatic p.M415I or p.M415V	Enhanced deubiquitinase activity	Corticotropinomas	Controversial: small tumors in one study, high rate of cavernous sinus invasion in a different study. Requires further evaluation	Potential, not yet tested: DUBs-IN (not available for clinical use)

See references in text. * It is not clear if any of the reported PitB cases have been caused by somatic variants. BP, branch point; DUBs-IN, deubiquitinase inhibitors; GTPase, guanosine triphosphatase; MEK, MAPK, and ERK kinase; NF-PitNETs, non-functioning pituitary neuroendocrine tumors; PitBs, pituitary blastomas; PitNETs, pituitary neuroendocrine tumors; RNase, ribonuclease; snRNP, small nuclear ribonucleoprotein.
